# Traumatic stress symptoms among Spanish healthcare workers during the COVID-19 pandemic: a prospective study

**DOI:** 10.1017/S2045796023000628

**Published:** 2023-08-09

**Authors:** Ana Portillo-Van Diest, Gemma Vilagut, Itxaso Alayo, Montse Ferrer, Franco Amigo, Benedikt L. Amann, Andrés Aragón-Peña, Enric Aragonès, Ángel Asúnsolo Del Barco, Mireia Campos, Isabel Del Cura-González, Meritxell Espuga, Ana González-Pinto, Josep M. Haro, Amparo Larrauri, Nieves López-Fresneña, Alma Martínez de Salázar, Juan D. Molina, Rafael M. Ortí-Lucas, Mara Parellada, José M. Pelayo-Terán, Aurora Pérez-Zapata, José I. Pijoan, Nieves Plana, Teresa Puig, Cristina Rius, Carmen Rodríguez-Blázquez, Ferran Sanz, Consol Serra, Iratxe Urreta-Barallobre, Ronald C. Kessler, Ronny Bruffaerts, Eduard Vieta, Víctor Pérez-Solá, Jordi Alonso, Philippe Mortier, MINDCOVID Working Group

**Affiliations:** 1 Health Services Research Unit, IMIM-Institut Hospital del Mar d’Investigacions Mèdiques, Barcelona, Spain; 2 CIBER de Epidemiología y Salud Pública, Instituto de Salud Carlos III, Madrid, Spain; 3 Asociación instituto de investigación en sistemas de salud Biosistemak, Barakaldo, País Vasco, España; 4 Department of Medicine and Life Sciences, Universitat Pompeu Fabra, Barcelona, Spain; 5 CIBER de Salud Mental, Instituto de Salud Carlos III, Madrid, Spain; 6 Centre Fórum Research Unit, Institute of Neuropsychiatry and Addictions (INAD), Parc de Salut Mar, Barcelona, Spain; 7 Department of Health Services Research Group, IMIM-Institut Hospital del Mar d’Investigacions Mèdiques, Barcelona, Spain; 8 Department for Psychiatry and Psychotherapy, Hospital of the Ludwig-Maximilians-University Munich, Germany; 9 Epidemiology Unit, Regional Ministry of Health, Community of Madrid, Madrid, Spain; 10 Fundación Investigación e Innovación Biosanitaria de AP, Comunidad de Madrid, Madrid, Spain; 11 Department of Atenció Primària Camp de Tarragona, Institut d’Investigació en Atenció Primària IDIAP Jordi Gol, Barcelona, Spain; 12 Atenció Primària Camp de Tarragona, Institut Català de la Salut, Spain; 13 Department of Surgery, Medical and Social Sciences, Faculty of Medicine and Health Sciences, University of Alcala, Alcalá de Henares, Spain; 14 Ramón y Cajal Institute of Sanitary Research (IRYCIS), Madrid, Spain; 15 Department of Epidemiology and Biostatistics, Graduate School of Public Health and Health Policy, The City University of New York, New York, NY, USA; 16 Service of Prevention of Labor Risks, Medical Emergencies System, Generalitat de Catalunya, Spain; 17 Research Unit, Primary Care Management, Madrid Health Service, Madrid, Spain; 18 Department of Medical Specialities and Public Health, King Juan Carlos University, Madrid, Spain; 19 Occupational Health Service, Hospital Universitari Vall d’Hebron, Barcelona, Spain; 20 BIOARABA, UPV-EHU, Hospital Universitario Araba-Santiago, Vitoria-Gasteiz, Spain; 21 Department of Research and Development Unit, Parc Sanitari Sant Joan de Déu, Barcelona, Spain; 22 Department Facultat de Medicina y Ciencias de la Salut, Universitat de Barcelona (UB), Barcelona, Spain; 23 National Center of Epidemiology, Instituto de Salud Carlos III, Madrid, Spain; 24 Department Medicina Preventiva, Hospital General Universitario Gregorio Marañón, Madrid, Spain; 25 UGC Salud Mental, Hospital Universitario Torrecárdenas, Almería, Spain; 26 Villaverde Mental Health Center, Clinical Management Area of Psychiatry and Mental Health, Psychiatric Service, Hospital Universitario 12 de Octubre, Madrid, Spain; 27 Research Institute Hospital 12 de Octubre (i+12), Madrid, Spain; 28 Faculty of Health Sciences, Universidad Francisco de Vitoria, Madrid, Spain; 29 Department of Preventive MedicineDepartment, Hospital Clínic Universitari, Valencia, Spain; 30 Servicio de Psiquiatría y Salud Mental, Hospital el Bierzo, Gerencia de Asistencia Sanitaria del Bierzo (GASBI), Gerencia Regional de Salud de Castilla y Leon (SACYL), Ponferrada, León, Spain; 31 Area de Medicina Preventiva y Salud Pública, Universidad de León, León, Spain; 32 Department Servicio de Prevención de Riesgos Laborales, Príncipe de Asturias University Hospital, Alcalá de Henares, Madrid, Spain; 33 Department Clinical Epidemiology Unit, Hospital Universitario Cruces/OSI EEC, Bilbao, Spain; 34 Department of Epidemiology and Public Health, Hospital de la Santa Creu i Sant Pau, Barcelona, Spain; 35 Biomedical Research Institute Sant Pau (IIB Sant Pau), Barcelona, Spain; 36 Department of Paediatrics, Obstetrics and Gynaecology and Preventive Medicine and Public HealthDepartment, Universitat Autònoma de Barcelona (UAB), Barcelona, Spain; 37 CIBER de Enfermedades Cardiovasculares, Instituto de Salud Carlos III, Madrid, Spain; 38 Agència de Salut Pública de Barcelona, Barcelona, Spain; 39 CIBER de Enfermedades Neurodegenerativas, Instituto de Salud Carlos III, Madrid, Spain; 40 Research Progamme on Biomedical Informatics (GRIB), Hospital del Mar Medical Research Institute (IMIM), Barcelona, Spain; 41 Instituto Nacional de Bioinformatica – ELIXIR-ES (IMPaCT-Data-ISCIII), Barcelona, Spain; 42 Departament de Psiquiatria i Medicina Legal, Parc de Salut Mar PSMAR, Barcelona, Spain; 43 CiSAL-Centro de Investigación en Salud Laboral, IMIM/UPF, Barcelona, Spain; 44 Osakidetza Basque Health Service, Donostialdea Integrated Health Organisation, Donostia University Hospital, Clinical Epidemiology Unit, San Sebastián, Spain; 45 Clinical Epidemiology, Biodonostia Health Research Institute, San Sebastián, Spain; 46 Department of Health Care Policy, Harvard Medical School, Boston, MA, USA; 47 Center for Public Health Psychiatry, Universitair Psychiatrisch Centrum, KU Leuven, Leuven, Belgium; 48 Hospital Clínic, Institute of Neuroscience, University of Barcelona, IDIBAPS, Barcelona, Catalonia, Spain

**Keywords:** COVID-19, health personnel, prospective cohort study, traumatic stress

## Abstract

**Aim:**

To investigate the occurrence of traumatic stress symptoms (TSS) among healthcare workers active during the COVID-19 pandemic and to obtain insight as to which pandemic-related stressful experiences are associated with onset and persistence of traumatic stress.

**Methods:**

This is a multicenter prospective cohort study. Spanish healthcare workers (*N* = 4,809) participated at an initial assessment (i.e., just after the first wave of the Spain COVID-19 pandemic) and at a 4-month follow-up assessment using web-based surveys. Logistic regression investigated associations of 19 pandemic-related stressful experiences across four domains (infection-related, work-related, health-related and financial) with TSS prevalence, incidence and persistence, including simulations of population attributable risk proportions (PARP).

**Results:**

Thirty-day TSS prevalence at T1 was 22.1%. Four-month incidence and persistence were 11.6% and 54.2%, respectively. Auxiliary nurses had highest rates of TSS prevalence (35.1%) and incidence (16.1%). All 19 pandemic-related stressful experiences under study were associated with TSS prevalence or incidence, especially experiences from the domains of health-related (PARP range 88.4–95.6%) and work-related stressful experiences (PARP range 76.8–86.5%). Nine stressful experiences were also associated with TSS persistence, of which having patient(s) in care who died from COVID-19 had the strongest association. This association remained significant after adjusting for co-occurring depression and anxiety.

**Conclusions:**

TSSs among Spanish healthcare workers active during the COVID-19 pandemic are common and associated with various pandemic-related stressful experiences. Future research should investigate if these stressful experiences represent truly traumatic experiences and carry risk for the development of post-traumatic stress disorder.

## Introduction

Healthcare workers (HCW) are at increased risk for traumatic experiences and subsequent development of traumatic stress symptoms (TSS; Skogstad *et al.*, 2013). According to the 5th edition of the Diagnostic and Statistical Manual of Mental Disorders or DSM-5 criteria (American Psychiatric Association, 2013), traumatic events are restricted to exposure to death, threatened death, actual or threatened serious injury or actual or threatened sexual violence. TSSs following traumatic events include intrusion (e.g., recurrent distressing memories of the traumatic event), negative mood (i.e., the persistent inability to experience positive emotions), dissociative symptoms (e.g., depersonalization and dissociative amnesia), avoidance (e.g., avoidance of memories, thoughts or feelings related to the traumatic event) and alterations in arousal (e.g., sleep disturbances, irritability and anger outbursts and hypervigilance) (Regier *et al.*, 2013). Symptoms typically begin immediately after the traumatic event, and persistence for at least 3 days is needed to meet the criteria for acute stress disorder (ASD). Once symptoms last longer than 1 month, the diagnosis of post-traumatic stress disorder can be considered. The pathophysiology of TSS is considered to be related to the failure to adapt to fear conditioning through extinction learning (Bryant, 2018), and neuroimaging studies have documented altered neural functioning in several brain regions (Cwik *et al.*, 2017; Geuze *et al.*, 2008).

There is concern that the COVID-19 pandemic has increased the risk for TSS among HCW, as recent meta-analyses found high prevalence rates (13–22%) of post-traumatic stress disorder (PTSD) symptoms among HCWs active during coronavirus outbreaks (Carmassi *et al.*, 2020; Salazar de Pablo *et al.*, 2020; Salehi *et al.*, 2021; Serrano-Ripoll *et al.*, 2020). The COVID-19 pandemic also questions prevailing PTSD models as these models only consider direct exposure to certain past life-threatening events as potentially traumatic, in line with strict DSM-5 diagnostic criteria (American Psychiatric Association, 2013; Husky *et al.*, 2021). Broader definitions of traumatic stressors also exist (World Health Organization, 2018), and in the context of this study, we assume that a prolonged exposure to intense stressors can also be experienced as traumatic and potentially yield TSS. This is in line with recent work (Bridgland *et al.*, 2021) that suggests that TSS could also be caused by anticipating future events, indirect exposure to a potentially traumatic event, as well as a broader range of stressful experiences than those complying with DSM-5’s strict definition of trauma. This highlights the need for prospective studies that use large representative samples to examine the associations of various pandemic-related stressors with TSS among HCW (Husky *et al.*, 2021). Prospective studies are also needed to provide more insight into the complex patterns of TSS through time, including resilient, delayed onset, recovering and chronically distressed patterns (Bryant, 2018, 2019). In the context of the COVID-19 pandemic, it is therefore important to carefully differentiate between TSS directly following pandemic-related stressful experiences, on the one hand, and onset or persistence of TSS later in time, on the other hand.

We address this need by presenting prospective data from a large probabilistic sample of Spanish HCW, recruited as part of the MINDCOVID project (Alonso *et al.*, 2021). Spain was among those countries hit particularly hard by the COVID-19 pandemic, especially during the first wave, which placed the Spanish healthcare system under extreme pressure (Pacchiarotti *et al.*, 2020). We aim to (1) quantify prevalence, incidence and persistence of TSS related to the first wave of the COVID-19 pandemic (March–July 2020); (2) investigate associations of a wide range of pandemic-related stressful experiences with TSS and (3) obtain preliminary estimates of the proportions of TSS that are potentially preventable through interventions.

## Materials and methods

### Study design, population and sampling

As part of the MINDCOVID project (Alonso *et al.*, 2021), a multicenter, observational, prospective cohort study of HCW was carried out, consisting of an initial assessment (T1) and a 4-month follow-up assessment (T2). For the initial assessment (May 5—September 7, 2020, i.e., just after the height of the first wave of the Spain COVID-19 pandemic; Supplementary Figure S1), HCWs were recruited from 18 healthcare institutions (six Spanish Autonomous Communities). Each centre contacted all employed workers using administrative email distribution lists (i.e., census sampling). Participants had to be currently employed and aged 18 years or older and were excluded if they were unable to understand the survey language or did not provide explicit consent. The invitation email included an anonymous link to the web‐based survey platform (Qualtrics.com). After opening the link, participants were provided with key information regarding the study and treatment of data and were asked if they had read all information and agreed to participate in the study. Two reminder emails were sent within a 2-to-4-week period after the initial invitation. A total of 8,996 HCWs participated in the T1 survey (response rate = 11.7%; May–September 7, 2020; Supplementary Figure S2). Of those, 4,809 (65.7% cooperation rate) participated in a follow-up survey (October 9–November 24, 2020), on average 4 months (120.1 days; SD = 22.2) after the initial assessment (Supplementary Figure S2). Sample characteristics slightly shifted between assessments, but the differences were very small (Supplementary Table S1). Inverse probability weighting (Seaman & White, 2011; Seaman *et al.*, 2012) was applied to account for these differences (see Statistical analysis section).

### Measures

#### Outcome variable

Thirty-day TSSs are defined as a positive screen on a four-item version (Zuromski *et al.*, 2019) of the PTSD checklist for DSM-5 or PCL-5 (Blevins *et al.*, 2015; Weathers *et al.*, 2013). Several short forms of the Posttraumatic Stress Disorder Checklist or PCL exist (Bliese *et al.*, 2008; Lang & Stein, 2005; Price *et al.*, 2016). The PCL-5 four-item version we used generates outcomes that most closely parallel those of the full PCL‐5 and has been developed using different statistical techniques (exploratory factor analysis, stepwise logistic regression and machine learning methods) to identify the optimal integer-scored short-form scale (Zuromski *et al.*, 2019). Items selected for this optimal scale assess intrusion (“Suddenly feeling or acting as if the stressful experience were actually happening again (as if you were actually back there reliving it)?”), avoidance (“Avoiding external reminders of the stressful experience (for example, people, places, conversations, activities, objects, or situations)?”), negative alterations in cognition and mood (“Feeling distant or cut off from other people?”) and alterations in arousal and reactivity (“Irritable behaviour, angry outbursts, or acting aggressively?”). Each of the items is scored from 0 to 4 (“Not at all” to “Extremely”), which are then summed to obtain one single (unidimensional) short-form scale score. With a scale score cutoff point of seven, the short-form predicts PCL-5 ≥ 28 diagnostic thresholds with AUC = 0.916, sensitivity = 85.5%, and specificity = 97.8% (for additional details, see Zuromski *et al.*, 2019). The official Spanish translation of the PCL-5 scale was provided to us through email by the US National Center for PTSD (https://www.ptsd.va.gov/) and coincides with the translation available at the official website for Cognitive Processing Therapy for PTSD (https://cptforptsd.com/cpt-resources/). We use the more generic term ‘traumatic stress symptoms’ instead of ASD or PTSD because we did not explicitly determine the presence of TSSs for more than 30 days (criterion F for PTSD according to the DSM-5) and because a short screening instrument was used to assess the outcome.

In line with insights into the complex course of traumatic stress following traumatic events (Bryant, 2018, 2019), we used three operationalizations of TSS in time: (1) TSS prevalence at T1 in order to provide insight into the onset of traumatic stress most proximate to the pandemic-related stressful experiences under study; (2) TSS incidence, defined as the proportion of respondents with a positive four-item PCL-5 screen at T2 among those with a negative four-item PCL-5 screen at T1 in order to provide insight in the delayed onset of traumatic stress; and (3) TSS persistence, defined as the proportion of respondents with a positive four-item PCL-5 screen at T2 among those with a positive four-item PCL-5 screen at T1 in order to provide insight into the development of chronic patterns of TSS.

#### Distal (pre-pandemic) risk factors

We considered 11 distal (i.e., pre-pandemic) risk factors, assessed in the initial survey: age, gender, country of birth, marital status, pre-pandemic monthly income, having children in care, type of profession, type of workplace, number of pre-pandemic lifetime mental disorders (assessed using a checklist based on the Composite International Diagnostic Interview; Kessler & Üstün, 2004), number of pre-pandemic physical health conditions (assessed using a seven-item checklist; Sangha *et al.*, 2003) and 12-month physical or sexual assault.

#### Proximal risk factors (pandemic-related stressful experiences)

In line with factors risk factors found in previous literature (Annaloro *et al.*, 2021; Carmassi *et al.*, 2020; X. Li *et al.*, 2021a; Serrano-Ripoll *et al.*, 2020; Yuan *et al.*, 2021), four domains of pandemic-related stressful experiences were assessed in the initial survey. The recall period for all experiences was since the beginning of the Spain COVID-19 pandemic. A first domain considered three COVID-19 infection–related experiences: (1) personal COVID-19 infection status, that is, having been hospitalized for COVID-19 infection or having had a positive COVID-19 test or medical COVID-19 diagnosis not requiring hospitalization, (2) having loved ones infected with COVID‐19 and (3) having been in isolation or quarantine because of COVID‐19.

The second domain included eight work-related stressful experiences: (1) average weekly hours worked, (2) changes in assigned functions, team or working location; (3) perceived lack of training for assigned tasks (0–4 scale score), (4) the frequency of direct exposure to COVID-19 patients during professional activity (0–4 scale score), (5) the perceived lack of healthcare centre preparedness (0–4 scale score), (6) perceived frequency of lack of protective equipment (0–4 scale score), (7) having to make decisions regarding prioritizing care among COVID‐19 patients (assessed among medical doctors and nurses), and (8) having patient(s) in care who died from COVID‐19 (assessed among all HCW involved in patient care).

A third domain consisted of six variables that measured health-related stress using 0–4 scale scores: (1) feeling of little control over getting infected or not, (2) fear of infecting loved ones, (3) family and friends’ degree of worry of getting infected through the HCW, (4) degree to which people avoided the HCW’s family because of the HCW’s job, (5) personal health-related stress and (6) stress related to the health of loved ones.

A fourth domain consisted of two financial factors: (1) having suffered a significant loss in personal or family income due to the COVID‐19 pandemic and (2) stress over one’s financial situation (0–4 scale score). For a detailed description of all measures, see Supplementary Methods.

#### Depression and anxiety

Depression and anxiety (included as covariates; see Statistical analysis section) were assessed using the Spanish version (Diez-Quevedo *et al.*, 2001) of the Patient Health Questionnaire (PHQ-8; Kroenke *et al.*, 2009) and the Spanish version (García-Campayo *et al.*, 2010) of the Generalized Anxiety Disorder scale (GAD-7; Spitzer *et al.*, 2006).

### Statistical analysis

Analyses were conducted with SAS System for Windows 9.4 (SAS Institute Inc., Cary, NC, USA) and R version 4.1.1 (R Core Team, 2021). Analyses were restricted to the 4,809 respondents who participated in both initial and follow-up surveys. Non-response and attrition bias were tackled by calculating sample weights through a raking and inverse probability weighting procedure that matches the final sample (*n* = 4,809) to (1) the target population of Spanish HCW in participating centrrs (*n* = 103,578) according to healthcare centre, gender, age and professional category (overall and within each healthcare centre); and (2) the full sample of T1 participants (*n* = 8,996) according to all T1 survey variables. Multivariable imputation by chained equations with 12 imputed datasets and 10 iterations per imputation was used to address the minimal problem of item-level missing data.

Differences in TSS prevalence, incidence and persistence across distal risk factors were assessed using the modified Rao-Scott Chi-squared test. Logistic regression was used to estimate the associations of distal risk factors and pandemic-related stressful experiences with TSS. Results are reported as odd ratios (ORs) with 95% confidence intervals (95% CI). Individual-level associations of distal risk factors with TSS were estimated using a multivariable model including all distal risk factors. Subsequently, individual- and population-level associations of each separate pandemic-related stressful experience with TSS were estimated, each time adjusting for all distal risk factors (considered covariates). All analyses were adjusted for time (i.e., week) of T1 survey participation in order to adjust for individual variations in follow-up time between the T1 and T2 assessments. Since causal relationships between the included pandemic-related stressful experiences are largely unknown, we refrained from constructing a fully adjusted multivariable model to avoid the risk of overadjustment bias (Schisterman *et al.*, 2009). Given concerns regarding the unknown discriminative validity of the PCL-5 versus general negative emotionality such as depression and anxiety (Bridgland *et al.*, 2021) and given the high degree of co-morbidity between TSS/PTSD and other mental disorders (Bryant, 2018), we repeated the analyses investigating associations between pandemic-related stressful experiences and TSS, additionally adjusting for co-occurring depression and anxiety, and presented these results in Supplementary Tables S2–S3.

Population-level associations, that is, population attributable risk proportions (PARP) and their standard errors (SE) were calculated using simulation methods based on logistic regression equations. A PARP is the proportion of the cumulative predicted value of an outcome statistically explained by specific predictor variables (Centers for Disease Control and Prevention, 2012). PARPs can be interpreted as the expected proportional reduction in TSS if the risk factors or the causal factors accounting for the risk factors were eradicated in the population. It is important to note that PARPs can sum to more than 100% because some individuals with more than one risk factor can have TSS prevented in more than one way, and the prevented TSS cases of these individuals could be counted more than once (Rowe *et al.*, 2004).

**Table 1.** Associations of distal (pre-pandemic) risk factors with traumatic stress symptoms (*n* = 4,809)

	Sample descriptives		T1 prevalence TSS^a^		Incidence TSS^b^		Persistence TSS^c^	
	*n*^d^	% (SE)^e^	% (SE)^e^	OR (95% CI)^f^	% (SE)^e^	OR (95% CI)^f^	% (SE)^e^	OR (95% CI)^f^
Total			22.1 (1.3)		11.6 (1.4)		54.2 (2.8)	
Age								
18−29 years	503	11.0 (1.9)	21.7 (2.7)	Ref	15.3 (2.2)	Ref	50.3 (5.3)	Ref
30−49 years	2,275	45.5 (1.6)	23.7 (1.8)	1.23 (0.81−1.88)	13.7 (1.7)	0.79 (0.49−1.27)	53.3 (3.3)	1.29 (0.80−2.06)
50 years or more	2,031	43.5 (2.5)	20.6 (1.8)	0.91 (0.59−1.40)	8.5 (1.6)	0.42 (0.26−0.67)*	56.4 (4.3)	1.24 (0.65−2.39)
*p*-value pooled modified Rao-Scott *χ*^2^ test^g^			0.328		<0.001*		0.622	
Gender								
Male	910	22.4 (1.3)	15.6 (1.3)	Ref	10.3 (1.5)	Ref	54.4 (4.2)	Ref
Female	3,899	77.6 (1.3)	24.0 (1.4)	1.42 (1.18−1.72)*	12.0 (1.5)	0.99 (0.70−1.40)	54.2 (3.0)	1.05 (0.74−1.50)
*p*-value pooled modified Rao-Scott *χ*^2^ test^g^			<0.001*		0.246		0.953	
Country of birth								
Spain	4,582	95.4 (0.5)	22.2 (1.4)	Ref	11.6 (1.4)	Ref	54.6 (2.7)	Ref
Other	227	4.6 (0.5)	21.4 (1.8)	1.01 (0.75−1.37)	10.7 (2.3)	0.81 (0.53−1.23)	46.6 (8.2)	0.83 (0.45−1.52)
*p*-value pooled modified Rao-Scott *χ*^2^ test^g^			0.723		0.652		0.329	
Marital status								
Married	2,537	52.7 (2.2)	21.1 (1.5)	Ref	10.5 (1.3)	Ref	54.4 (2.4)	Ref
Single, divorced, legally separated or widowed	2,272	47.3 (2.2)	23.3 (1.4)	0.89 (0.74−1.07)	12.9 (2.0)	0.93 (0.67−1.31)	54.0 (4.3)	0.87 (0.61−1.26)
*p*-value pooled modified Rao-Scott *χ*^2^ test^g^			0.125		0.201		0.914	
Pre-pandemic monthly income								
Less than 2,200€	1,420	35.1 (2.2)	27.5 (1.7)	Ref	14.9 (2.4)	Ref	55.8 (3.9)	Ref
Between 2,200€ and 4,500€	1,730	36.1 (1.2)	20.8 (1.9)	0.79 (0.64−0.97)*	11.3 (1.8)	0.78 (0.52−1.17)	55.5 (4.8)	0.90 (0.63−1.28)
More than 4,500€	1,659	28.8 (1.5)	17.3 (1.2)	0.82 (0.65−1.04)	8.4 (1.2)	0.70 (0.51−0.96)*	49.3 (4.6)	0.75 (0.45−1.24)
*p*-value pooled modified Rao-Scott *χ*^2^ test^g^			<0.001*		0.010*		0.557	
Having children in care								
No	2,814	58.8 (1.4)	22.9 (1.6)	Ref	11.6 (1.4)	Ref	56.2 (4.0)	Ref
Yes	1,995	41.2 (1.4)	21.1 (1.6)	0.86 (0.72−1.01)	11.5 (1.6)	0.98 (0.72−1.33)	51.1 (3.9)	0.75 (0.46−1.21)
*p*-value pooled modified Rao-Scott *χ*^2^ test^g^			0.316		0.949		0.362	
Type of profession								
Medical doctor	1,650	26.3 (2.8)	12.4 (1.1)	Ref	8.7 (1.4)	Ref	49.1 (4.4)	Ref
Nurse	1,406	31.0 (1.2)	25.7 (1.9)	2.31 (1.93−2.77)*	12.5 (1.9)	1.57 (1.15−2.13)*	57.4 (4.0)	1.44 (0.86−2.41)
Auxiliary nurse	387	13.7 (3.2)	35.1 (3.5)	3.51 (2.61−4.72)*	16.1 (2.9)	2.76 (1.64−4.64)*	53.5 (7.2)	1.23 (0.60−2.54)
Other profession involved in patient care	555	9.0 (0.8)	17.0 (2.4)	1.39 (1.09−1.77)*	13.7 (2.4)	1.77 (0.89−3.51)	57.5 (4.6)	1.41 (0.74−2.69)
Other profession not involved in patient care	812	20.0 (2.4)	23.0 (3.0)	2.02 (1.38−2.95)*	10.9 (3.0)	1.44 (1.09−1.90)*	52.0 (4.7)	0.96 (0.61−1.52)
*p*-value pooled modified Rao-Scott *χ*^2^ test^g^			<0.001*		0.116		0.711	
Type of workplace								
Hospital (ED)	620	11.9 (0.6)	19.6 (2.2)	Ref	12.8 (1.8)	Ref	52.6 (5.7)	Ref
Hospital (not ED)	2,198	45.6 (0.9)	24.7 (1.6)	1.19 (0.93−1.53)	9.9 (1.7)	0.78 (0.49−1.24)	52.0 (4.1)	0.98 (0.55−1.73)
Primary care	1,581	36.2 (0.9)	21.1 (1.6)	1.22 (0.87−1.72)	14.4 (1.5)	1.54 (0.91−2.60)	59.7 (2.9)	1.51 (0.93−2.46)
Others	410	6.3 (0.4)	14.4 (2.5)	0.63 (0.42−0.94)*	5.3 (2.2)	0.34 (0.13−0.88)*	39.9 (6.6)	0.65 (0.29−1.47)
*p*-value pooled modified Rao-Scott *χ*^2^ test^g^			0.006*		0.021*		0.171	
Number of pre-pandemic lifetime mental disorders								
None	2,882	58.8 (0.9)	16.5 (1.6)	Ref	8.4 (1.0)	Ref	51.5 (3.2)	Ref
Exactly one	1,550	32.8 (0.9)	27.1 (1.7)	1.86 (1.41−2.46)*	16.0 (1.9)	1.96 (1.67−2.30)*	53.8 (3.8)	1.16 (0.85−1.58)
Two or more	377	8.4 (0.7)	42.2 (3.9)	3.46 (2.42−4.94)*	22.4 (5.3)	3.17 (1.98−5.06)*	62.7 (5.3)	1.68 (1.13−2.50)*
*p*-value pooled modified Rao-Scott *χ*^2^ test^g^			<0.001*		<0.001*		0.115	
Number of pre-pandemic physical health conditions								
None	3,639	74.1 (1.0)	20.8 (1.2)	Ref)	10.6 (1.2)	Ref)	51.7 (3.1)	Ref)
Exactly one	1,013	22.0 (0.8)	24.4 (2.5)	1.24 (0.97−1.57)	14.9 (2.4)	1.58 (1.22−2.06)*	57.2 (2.5)	1.25 (0.91−1.72)
Two or more	157	3.9 (0.5)	34.3 (5.6)	1.87 (1.06−3.32)*	12.6 (3.1)	1.11 (0.64−1.94)	70.9 (10.3)	2.39 (1.22−4.69)*
*p*-value pooled modified Rao-Scott *χ*^2^ test^g^			0.029*		0.018*		0.037*	
Twelve-month physical or sexual assault								
No	4,748	98.7 (0.2)	21.9 (1.2)	Ref	11.5 (1.4)	Ref	54.1 (2.9)	Ref
Yes	61	1.3 (0.2)	39.1 (8.4)	2.34 (1.17−4.70)*	25.2 (6.1)	2.62 (0.99−6.90)	58.7 (14.2)	1.42 (0.31−6.47)
*p*-value pooled modified Rao-Scott *χ*^2^ test^g^			0.046*		0.017*		0.745	

Abbreviations: CI = confidence interval; ED = emergency department; OR = odds ratio; SE = standard error; TSS = traumatic stress symptoms.

a Prevalence of TSS is defined as a positive screen on the four-item PCL-5 at T1 (*n* = 4,809).

b Incidence of TSS is defined as the proportion of respondents with a positive four-item PCL-5 screen at 4-month follow-up (*n* = 412) among those with a negative four-item PCL-5 screen at T1 (*n* = 3,796).

c Persistence of TSS is defined as the proportion of respondents with a positive four-item PCL-5 screen at 4-month follow-up (*n* = 536) among those with a positive four-item PCL-5 screen at T1 (*n* = 1,013).

d Number of observations (*n*) are unweighted.

e Proportions (%, SE) are weighted.

f Results represent one logistic regression model including all distal risk factors, additionally adjusting for time (i.e., week) of T1 survey participation.

g *p*-value based on a *F* test to evaluate statistical significance based on multiple imputations, using the procedure by Li et al. (1991)

* Indicate statistically significant results (*α* = 0.05).

## Results

### Individual-level associations of pre-pandemic (distal) risk factors with TSS

Thirty-day prevalence of TSS at T1 was estimated at 22.1%; 4-month incidence and persistence of TSS were estimated at 11.6% and 54.2%, respectively. TSS estimates stratified by distal risk factors and adjusted associations of distal risk factors with TSS are shown in Table 1. Compared to medical doctors, all other professional categories had higher odds for both TSS prevalence and incidence, especially auxiliary nurses and nurses. A history of pre-pandemic mental disorders was also strongly associated with prevalence and incidence of TSS, but persistent TSS was only significantly associated with having two or more lifetime mental disorders. A similar, although less consistent pattern was found for pre-pandemic physical health conditions. No clear associations were found for age, gender, and income, although results suggest higher TSS incidence among younger HCW, higher TSS prevalence among females, and higher odds for TSS onset among those with lower pre-pandemic monthly income levels. No associations were found for the country of birth, marital status, and having children in care with TSS.

**Table 2.** Associations of pandemic-related stressful experiences with traumatic stress symptoms (*n* = 4,809)

	Sample descriptives		T1 prevalence TSS^a^	Incidence TSS^b^	Persistence TSS^c^
	*n*^d^	% (SE) or Med (SE) (IQR)^e^	OR (95% CI)^f^	OR (95% CI)^f^	OR (95% CI)^f^
*A. COVID-19 infection*–*related stressful experiences*					
Personal COVID-19 infection status					
Never infected with COVID-19	3,873	82.5 (2.1)	Ref	Ref	Ref
Positive COVID-19 test or medical COVID-19 diagnosis without hospitalization	869	16.1 (2.0)	1.05 (0.87−1.27)	1.47 (1.16−1.88)*	1.25 (0.97−1.60)
Having been hospitalized for COVID-19	67	1.4 (0.2)	1.55 (0.71−3.41)	2.13 (0.86−5.25)	0.87 (0.25−3.04)
Having loved ones infected with COVID-19					
No loved ones infected	1,065	27.7 (2.4)	Ref	Ref	Ref
Partner, children or parents infected	781	13.7 (2.0)	1.32 (1.02−1.72)*	1.46 (1.02−2.09)*	1.45 (1.04−2.02)*
Other family, friends or others infected	2,964	58.6 (0.9)	1.00 (0.83−1.21)	1.03 (0.72−1.48)	1.20 (0.84−1.71)
Having been in isolation or quarantine because of COVID-19					
No	3,472	74.4 (1.7)	Ref	Ref	Ref
Yes	1,337	25.6 (1.7)	1.17 (1.02−1.33)*	1.76 (1.34−2.30)*	1.51 (1.08−2.13)*
*B. Work-related stressful experiences*					
Average weekly hours worked					
40 hours or less	2,905	63.2 (2.5)	Ref	Ref	Ref
41−50 hours	1,140	22.7 (2.4)	1.52 (1.25−1.84)*	0.97 (0.69−1.38)	1.13 (0.81−1.58)
51 hours or more	765	14.0 (0.7)	1.74 (1.34−2.25)*	1.02 (0.69−1.50)	0.96 (0.61−1.51)
Changes in assigned functions, team or working location					
No changes	2,088	45.3 (1.6)	Ref	Ref	Ref
Change of team or assigned functions	1,642	33.7 (3.2)	1.49 (1.21−1.83)*	1.32 (0.86−2.04)	1.55 (1.06−2.28)*
Changed to specific COVID-19 related work location	1,080	21.1 (3.7)	1.73 (1.36−2.18)*	1.53 (0.97−2.40)	1.42 (0.91−2.23)
Perceived lack of training for assigned tasks (scale 0−4)		0.7 (0.1) (0.0−1.7)	1.55 (1.40−1.72)*	1.19 (1.07−1.33)*	1.21 (1.04−1.41)*
Frequency of direct exposure to COVID-19 patients during professional activity (scale 0−4)		1.8 (0.2) (1.0−2.9)	1.42 (1.25−1.61)*	1.37 (1.19−1.59)*	1.28 (1.11−1.47)*
Perceived lack of healthcare centre preparedness (scaled 0−4)		1.6 (0.1) (0.7−2.4)	1.79 (1.58−2.02)*	1.61 (1.41−1.83)*	1.30 (1.10−1.53)*
Perceived frequency of lack of protective equipment (scale 0−4)		1.7 (0.1) (1.0−2.5)	1.77 (1.58−1.98)*	1.32 (1.18−1.48)*	1.11 (0.92−1.35)
Having to make decisions regarding prioritizing care among COVID−19 patients					
No	3,919	84.2 (1.8)	Ref	Ref	Ref
Yes	890	15.8 (1.8)	1.65 (1.35−2.00)*	1.68 (1.16−2.44)*	1.27 (0.90−1.77)
Having patient(s) in care that died from COVID-19					
No	2,882	62.8 (3.2)	Ref	Ref	Ref
Yes	1,927	37.2 (3.2)	1.29 (1.04−1.59)*	1.37 (0.97−1.93)	1.66 (1.16−2.38)*
*C. Health-related stressful experiences*					
Feeling of little control of getting infected or not (scale 0−4)		1.7 (0.1) (1.0−2.5)	1.90 (1.71−2.11)*	1.45 (1.30−1.63)*	1.12 (0.90−1.39)
Fear of infecting loved ones (scale 0−4)		2.8 (0.1) (1.7−3.4)	1.80 (1.55−2.10)*	1.76 (1.51−2.05)*	1.21 (0.93−1.59)
Family and friends’ degree of worry of getting infected through the HCW (scale 0−4)		1.5 (0.1) (0.5−2.4)	1.58 (1.47−1.70)*	1.33 (1.18−1.51)*	1.05 (0.88−1.25)
Degree to which people avoided the HCW’s family because of the HCW’s job (scale 0−4)		0.0 (0.0) (0.0−0.4)	1.58 (1.48−1.70)*	1.36 (1.22−1.52)*	1.23 (0.99−1.53)
Personal health-related stress (scale 0−4)		1.6 (0.1) (0.8−2.4)	2.48 (2.20−2.78)*	1.74 (1.56−1.95)*	1.30 (1.10−1.53)*
Stress related to the health of loved ones (scale 0−4)		2.4 (0.1) (1.5−3.2)	2.66 (2.35−3.02)*	1.84 (1.66−2.04)*	1.57 (1.19−2.07)*
*D. Financial stressful experiences*					
Significant loss of personal or familial income due to COVID-19					
No	3,924	79.8 (1.2)	Ref	Ref	Ref
Yes	885	20.2 (1.2)	1.63 (1.24−2.15)*	1.66 (1.13−2.44)*	1.11 (0.66−1.88)
Financial stress (scale 0−4)		0.6 (0.0) (0.0−1.6)	1.49 (1.40−1.59)*	1.28 (1.12−1.47)*	1.11 (0.97−1.28)

Abbreviations: CI = confidence interval; ED = emergency department; IQR = interquartile range; Med = median; OR = odds ratio; SE = standard error; TSS = traumatic stress symptoms.

a Prevalence of TSS is defined as a positive screen on the 4-item PCL-5 at T1 (*n* = 4,809).

b Incidence of TSS is defined as the proportion of respondents with a positive four-item PCL-5 screen at 4-month follow-up (*n* = 412) among those with a negative four-item PCL-5 screen at T1 (*n* = 3,796).

c Persistence of TSS is defined as the proportion of respondents with a positive four-item PCL-5 screen at 4-month follow-up (*n* = 536) among those with a positive four-item PCL-5 screen at T1 (*n* = 1,013).

d Number of observations (*n*) are unweighted.

e Proportions (%, SE) and Medians (SE) (IQR) are weighted.

f Each row represents a separate logistic regression model, each time adjusting for all distal risk factors and time (i.e., week) of T1 survey participation.

* Indicate statistically significant results (*α* = 0.05).

### Individual-level associations of pandemic-related stressful experiences with TSS

Adjusted associations of pandemic-related stressful experiences with TSS are shown in Table 2. Higher odds for all TSS outcomes under study were found among those with a COVID-19 infection of their partner, child, or parent, and among those who had been in isolation or quarantine for COVID-19. Those who tested positive for COVID-19 or ever had an established diagnosis of COVID-19 had significantly higher odds for TSS incidence only. All the eight work-related stressful experiences under study were significantly associated with TSS prevalence at T1. Five out of eight were also associated with TSS incidence, especially having to make decisions regarding prioritization of care and perceived lack of healthcare centre preparedness. Odds for persistent TSS were significantly higher among those who had a patient in care who died of COVID, those who had to change teams or functions, followed by perceived lack of healthcare centre preparedness, perceived lack of training for assigned tasks, and frequency of direct exposure to COVID-19 patients. All six health-related stressful experiences were found to be associated with both TSS prevalence and incidence, while personal health-related stress and stress related to the health of loved ones were also associated with persistence of TSS. Finally, loss of personal or familial income due to the pandemic and financial stress were associated with both TSS prevalence and incidence but not persistence of TSS.

### Population-level associations between pandemic-related stressful experiences and TSS

Adjusted PARP estimates for the associations of pandemic-related stressful experiences with TSS are shown in Table 3. Two stressful experience domains were consistently associated with all three TSS outcomes, most strongly with TSS prevalence and incidence: health-related and work-related stressful experiences. Within these domains, consistently high PARP was found for frequency of direct exposure to COVID-19 patients, perceived lack of healthcare centre preparedness, personal health-related stress, stress related to the health of loved ones and fear of infecting loved ones, with PARP in the range of 48.5–93.4% for TSS prevalence and incidence and in the range of 28.3–60.3% for TSS persistence. Financial factors were associated with both TSS prevalence and incidence, while infection-related experiences were only weakly associated with TSS incidence.

**Table 3.** Population attributable risk proportions for the associations of pandemic-related stressful experiences with TSS (*n* = 4,809)

	T1 prevalence TSS^a^	Incidence TSS^b^	Persistence TSS^c^
	% (SE)^d^^,^^e^	% (SE)^d^^,^^e^	% (SE)^d^^,^^e^
A. COVID-19 infection–related stressful experiences			
Personal COVID-19 infection	1.2 (1.7)	7.4 (2.6)*	1.6 (1.9)
Having loved ones infected with COVID-19	3.5 (6.3)	6.9 (9.5)	7.7 (7.3)
Having been in isolation or quarantine because of COVID-19	3.2 (2.1)	14.3 (3.4)*	5.5 (2.3)*
Risk domain A: total PARP^f^	5.1 (6.7)	17.6 (8.7)*	11.1 (7.7)
B. Work-related stressful experiences			
Average weekly hours worked	13.6 (2.9)*	−0.2 (4.2)	1.1 (3.6)
Changes in assigned functions, team or working location	18.5 (4.1)*	15.7 (6.4)*	11.3 (5.0)*
Perceived lack of training for assigned tasks (scale 0−4)	40.3 (3.1)*	18.8 (5.4)*	15.4 (5.6)*
Frequency of direct exposure to COVID-19 patients during professional activity (scale 0−4)	49.0 (4.9)*	48.5 (6.8)*	28.3 (9.2)*
Perceived lack of healthcare centre preparedness (rescaled 0−4)	65.5 (3.7)*	60.1 (6.0)*	28.4 (8.6)*
Perceived frequency of lack of protective equipment (scale 0−4)	66.6 (3.4)*	41.1 (7.3)*	12.5 (9.1)
Having to make decisions regarding prioritizing care among COVID-19 patients	6.3 (1.7)*	8.1 (2.7)*	2.2 (2.2)
Having patient(s) in care that died from COVID-19	7.3 (3.1)*	10.5 (5.0)*	10.0 (3.7)*
Risk domain B: total PARP^f^	86.5 (2.1)*	76.8 (5.2)*	47.6 (10.6)*
C. Health-related stressful experiences			
Feeling of little control of getting infected or not (scale 0−4)	72.0 (3.5)*	51.8 (7.1)*	13.4 (9.9)
Fear of infecting loved ones (scale 0−4)	80.3 (3.9)*	80.2 (4.5)*	29.1 (13.7)*
Family and friends’ degree of worry of getting infected through the HCW (scale 0−4)	54.6 (4.4)*	38.8 (6.8)*	4.9 (7.9)
Degree to which people avoided the HCW’s family because of the HCW’s job (scale 0−4)	19.4 (2.1)*	12.8 (3.0)*	8.0 (3.1)*
Personal health–related stress (scale 0−4)	83.8 (2.0)*	64.6 (4.9)*	30.4 (9.7)*
Stress related to the health of loved ones (scale 0−4)	93.4 (1.3)*	80.4 (4.4)*	60.3 (10.8)*
Risk domain C: total PARP^f^	95.6 (1.1)*	88.4 (3.1)*	58.4 (12.6)*
D. Financial stressful experiences			
Significant loss of personal or familial income due to COVID-19	7.2 (1.9)*	8.3 (2.9)*	1.1 (2.3)
Financial stress (scale 0−4)	34.9 (3.3)*	23.3 (5.1)*	7.7 (5.3)
Risk domain D: total PARP^f^	34.9 (3.3)*	23.4 (5.1)*	7.7 (5.2)

Abbreviations: COVID-19 = coronavirus disease 2019; SE = standard error; TSS = traumatic stress symptoms.

a Prevalence of TSS is defined as a positive screen on the four-item PCL-5 at T1 (*n* = 4,809).

b Incidence of TSS is defined as the proportion of respondents with a positive four-item PCL-5 screen at 4-month follow-up (*n* = 412) among those with a negative four-item PCL-5 screen at T1 (*n* = 3,796).

c Persistence of TSS is defined as the proportion of respondents with a positive four-item PCL-5 screen at 4-month follow-up (*n* = 536) among those with a positive four-item PCL-5 screen at T1 (*n* = 1,013).

d Proportions (%, SE) are weighted.

e Each row represents a separate logistic regression model, each time adjusting for all distal risk factors and time (i.e., week) of T1 survey participation.

f Risk domain total PARPs are based on four separate logistic regression models, one for each of the four proximal risk factor domains (A–D). Each model includes the proximal risk factors from the corresponding proximal risk factor domain (but not the other domains), adjusting for all distal risk factors and time (i.e., week) of T1 survey participation.

* Statistically significant results (*α* = 0.05).

### Analyses additionally adjusting for co-occurring depression and anxiety

We re-estimated all individual-level and population-level associations between pandemic-related stressful experiences and TSS, additionally adjusting for co-occurring depression (PHQ-8 total score) and anxiety (GAD-7 total score; see Supplementary Tables S2–3). Main findings from these analyses were as follows: (1) none of the associations between COVID-19 infection–related experiences and TSS remained significant, (2) having a patient in care die of COVID-19 was the only experience that remained significantly associated with TSS persistence (OR = 1.86; PARP = 8.0%), (3) all of the other stressful experiences remained significantly associated either with TSS prevalence or TSS incidence, except having to make decisions regarding prioritizing care among COVID-19 patients. While PARP estimates for the associations of the domains of health-related and work-related stressful experiences with TSS prevalence and incidence decreased considerably in strength (i.e., with a factor 1.5–1.9), these PARP were still in the range of 45.5–59.1%.

## Discussion

Prevalence of TSSs among HCWs active during the first wave of the Spanish COVID-19 pandemic is estimated at 22.1%, and incidence and persistence of TSS 4 months later into the pandemic is estimated at 11.6% and 54.2%, respectively. While almost all of the pandemic-related stressors under study were associated with TSS onset (i.e., either prevalence or incidence of TSS) to some extent, considerably less were associated with persistence of TSS.

Our T1 TSS prevalence estimate of 22.1% is in line with pooled estimates found in meta-analyses of post-TSSs among HCWs active during coronavirus outbreaks (13–22%; Y. Li *et al.*, 2021b; Salazar de Pablo *et al.*, 2020; Salehi *et al.*, 2021; Serrano-Ripoll *et al.*, 2020) and higher than the prevalence of 15.8% estimated in the Spanish general population during the pandemic (González-Sanguino *et al.*, 2020). Nurses, especially auxiliary nurses, were found to be the most vulnerable medical profession for developing TSS, with 2.3–3.5 times higher odds for TSS prevalence and 1.6–2.8 times higher odds for TSS incidence, compared to medical doctors, highlighting the negative impact of the COVID-19 pandemic on nurses’ mental well-being (Maben *et al.*, 2022). It is not possible to compare estimates of our prospective TSS outcomes with other HCW populations since very few previous studies present longitudinal data (Baumann *et al.*, 2007; Cai *et al.*, 2020; Canal-Rivero *et al.*, 2022; Jordan *et al.*, 2021; Yamane *et al.*, 2022) and none calculated incidence or persistence rates. An important finding from our study is therefore that just over half of HCW with significant TSS (i.e., 12% in total) continue to experience TSS 4 months later. This highlights the fact that PTSD estimates from previous studies among HCW may be inflated by transient stress reactions and ASD due to the exclusive use of cross-sectional surveys without follow-up assessments. Nevertheless, our estimate of 12% of HCW with persistent TSS is still considerably higher than reliable lifetime PTSD diagnosis estimates in the pre-pandemic general Spanish population (2.2–4.5%; Koenen *et al.*, 2017). Further monitoring of our HCW cohort is therefore warranted in order to detect the onset of PTSD symptoms, especially since many cases of PTSD do not present ASD symptoms directly following the traumatic experience and symptoms of PTSD may fluctuate over time (Bryant, 2018).

An important contribution from our study is that we investigated associations of a wide range of pandemic-related stressful experiences with TSS. Of all domains under study, we found that health-related stressful experiences were consistently the ones with the strongest association with all three operationalizations of TSS in time (i.e., prevalence, incidence and persistence). This is in line with previous studies using cross-sectional study designs that found that traumatic stress was associated with fear of infecting others (Bayazit *et al.*, 2022; Billings *et al.*, 2021; Greene *et al.*, 2020; Norful *et al.*, 2021), having family members infected (Al Falasi *et al.*, 2021; Blanco-Daza *et al.*, 2022), stress over one’s own health and feeling little control over getting infected (Annaloro *et al.*, 2021; Blanco-Daza *et al.*, 2022; Johnson *et al.*, 2020; Luceño-Moreno *et al.*, 2020; Ouyang *et al.*, 2022; Si *et al.*, 2020). We confirm these previous findings, and our study now suggests that health-related stressful experiences were also associated with delayed onset as well as persistence of TSS among HCW following the first COVID-19 outbreak in Spain. Work-related stressful experiences were also strongly associated with TSS, especially perceived lack of centre preparedness and lack of protective equipment, both of which have been extensively described in the literature as important risk factors (Annaloro *et al.*, 2021; D’Ettorre *et al.*, 2021; Norful *et al.*, 2021; Serrano-Ripoll *et al.*, 2020). Of note, we also found that having been in isolation or quarantine due to COVID had a significant association with TSS incidence, in line with findings from previous cross-sectional studies (Carmassi *et al.*, 2020; Pan *et al.*, 2021; Serrano-Ripoll *et al.*, 2020; Yuan *et al.*, 2021).

While our findings suggest that almost all of the pandemic-related stressful experiences under study are able to provoke traumatic stress to some extent, only a few were predictive of TSS persistence. The risk factor with the highest association with persisting TSS in our study was having patient(s) who died from COVID, in line with previous cross-sectional findings (Bayazit *et al.*, 2022; Leng *et al.*, 2021; Lockett *et al.*, 2022). Interestingly, this was also the only stressful experience under study considered consistent with DSM-5 criterion A of PTSD and the only one consistently associated with TSS persistence in all analyses (i.e., even after additionally adjusting for co-occurring depression and anxiety). This highlights the need for further empirical evidence in order to settle the ongoing debate on which traumatic experiences could be considered, triggering events for a PTSD diagnosis in the context of the COVID-19 pandemic (Bridgland *et al.*, 2021; Husky *et al.*, 2021).

### Limitations

Some limitations of our study need to be considered. First, TSS were measured using a self-reported four-item version of the PCL-5 and not through face-to-face clinical diagnosis. In addition, the time frame of TSS assessment only spans the past 30 days; hence, it is possible that persistent cases did not experience TSS during all 4 months between T1 and T2 assessment, and incident cases may include reactivation of TSS experienced earlier. The latter was partly addressed by adjusting all analyses for pre-pandemic mental disorders. Second, although we established temporality in our study, we did not ask respondents which specific stressful experience(s) provoked TSS, and we cannot exclude that the stressful experiences identified as significant in the analyses are mere markers of co-occurring truly traumatic events (assessed or not assessed in our study). Third, although we included a wide range of pandemic-related stressful experiences in our study, important stressful experiences may have been missed. Fourth, T1 participation in our study was low, however, in line with the pooled response rate of 13.0% among HCW web-based surveys worldwide (Cho *et al.*, 2013). We improved representativeness of our data by including census sampling and state-of-the-art missing data handling techniques to minimize selection bias. Finally, HCW who were sick during assignments may not have participated in the study, potentially causing selection bias.

## Conclusions

Onset and persistence of TSS among Spanish HCWs active during the first wave of the COVID-19 pandemic was high. TSS onset was especially high among nurses and auxiliary nurses and associated with a wide range of pandemic-related stressful experiences, including direct COVID-19 infection–related events, as well as health-related, work-related and financial factors. It remains unclear to what extent these experiences have intrinsic traumatic stress-provoking potential if they represent risk factors for subsequent PTSD onset or if they simply represent different markers for a prolonged period of time associated with traumatic stress. Future research should delineate etiological causal frameworks for the onset of TSS and PTSD. Such frameworks could subsequently guide prevention interventions and hereby address the absolute lack of research on effective interventions for mental disorders among healthcare personnel, both on the individual and on the organizational level (Petrie *et al.*, 2019).

Our study suggests that interventions tackling the onset of traumatic stress during viral outbreaks should primarily increase healthcare centre preparedness for viral outbreaks in order to prevent unexpected work-related changes, moral distress and health-related stress. Furthermore, due to the variability of TSS across time, there needs to be a clear assessment of trauma-related emotional distress at different timepoints of a crisis. This will allow to identify individuals who need help, as well as prevent development of persistent TSS (Bryant, 2018; Fanai & Khan, 2022). Healthcare centres’ preparedness should be done through bettering equipment, human resources, training and protocols. It is therefore encouraging that the International Labor Organization and the World Health Organization have recently published a guide on developing and implementing stronger occupational health and safety programs for health workers, focusing on various occupational hazards, including infectious and psycho-social hazards (World Health Organization, & International Labour Organization, 2022). The COVID‐19 pandemic has highlighted the importance of a well‐functioning healthcare system. Improving future mental health and promoting fair financial and working conditions among HCW should therefore be an absolute priority.

## Data Availability

The de-identified participant data as well as the study protocol and statistical analysis plan used for this study are available upon reasonable request from the corresponding authors (P.M. and J.A.) as long as the main objective of the data sharing request is replicating the analysis and findings as reported in this paper.

## Supplementary material

For supplementary material accompanying this paper visit http://doi.org/10.1017/S2045796023000628. click here to view supplementary material
