# Supplementary material for: Traumatic stress symptoms among Spanish healthcare workers during the COVID-19 pandemic: a prospective study
Source: Epidemiol Psychiatr Sci. 2023 Aug 9;32:e50. doi: 10.1017/S2045796023000628 (PMC10465320; doi:10.1017/S2045796023000628)

**Supplementary Figure 1. Daily new cases and timing of baseline and follow-up assessments.**


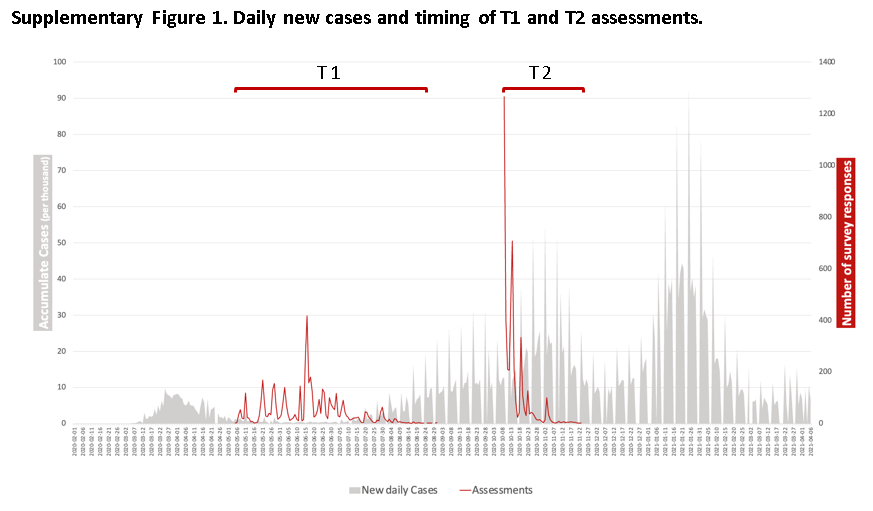


**Supplementary Figure 2. Diagram of recruitment process.**


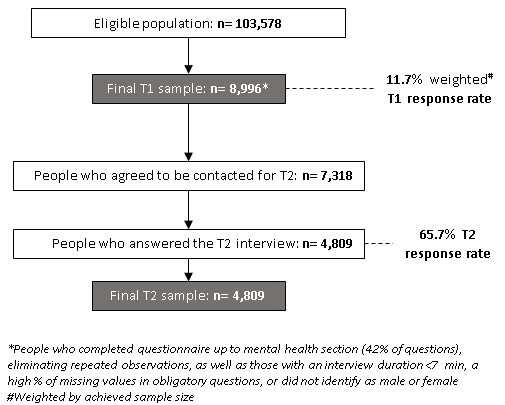

Supplement: Supplementary file 1 [file epssup.zip › S2045796023000628sup001.docx]
