# Supplementary material for: Traumatic stress symptoms among Spanish healthcare workers during the COVID-19 pandemic: a prospective study
Source: Epidemiol Psychiatr Sci. 2023 Aug 9;32:e50. doi: 10.1017/S2045796023000628 (PMC10465320; doi:10.1017/S2045796023000628)
Supplement: Supplementary file 1 [file epssup.zip › S2045796023000628sup002.docx]

**Materials and methods - Measures**

**Outcome variable**

**Thirty-day traumatic stress symptoms** (TSS) are defined as a positive screen on the four-item version (Zuromski et al., 2019) of the PTSD checklist for DSM-5 or PCL-5 (Blevins et al., 2015; Weathers et al., 2013) using a cut-off point of seven (Zuromski et al., 2019). The official Spanish translation of the PCL-5 scale was provided to us through email by the U.S. National Center for PTSD (<https://www.ptsd.va.gov/>), and coincides with the translation available at the official website for Cognitive Processing Therapy for PTSD (<https://cptforptsd.com/cpt-resources/>). This four-item version generates outcomes that closely parallel those of the full PCL‐5 (AUC>0.9; Zuromski et al., 2019). We use the more generic term “traumatic stress symptoms (TSS)” instead of Acute Stress Disorder (ASD) or Post Traumatic Stress Disorder (PTSD), because we did not explicitly determine presence of TSS for more than 30-days (criterion F for PTSD according to the DSM-5), and because a short screening instrument was used to assess the outcome.

**Description** of the four-item version of the PCL-5:

- Introduction: “*Below is a list of problems that people sometimes have in response to a very stressful experience. This stressful event can be related to the ongoing COVID-19 pandemic (for example, severe illness or death of a loved one, intense stress at work, having been isolated or in quarantine, …) or can be any other stressful event you once experienced. Please read each problem carefully and then indicate how much you have been bothered by that problem in the past 30 days*”.
- Response options: “*Not at all*”, “*A little bit*”, “*Moderately*”, “*Quite a bit*”, “*Extremely*”.
- List of traumatic stress symptoms: “*Suddenly feeling or acting as if the stressful experience were actually happening again (as if you were actually back there reliving it)?*”, “*Avoiding external reminders of the stressful experience (for example, people, places, conversations, activities, objects, or situations)?*”, “*Feeling distant or cut off from other people?”, “Irritable behavior, angry outbursts, or acting aggressively?*”

Given concerns regarding the unknown discriminative validity of the PCL-5 versus general negative emotionality such as depression and anxiety (Bridgland et al. 2021) and given the high degree of co-morbidity between TSS/PTSD and other mental disorders (Bryant, 2018), we conducted analyses investigating associations between pandemic-related stressful experiences and TSS, additionally adjusting for co-occurring depression and anxiety. **Depression and anxiety** were assessed using the Patient Health Questionnaire (PHQ-8; Kroenke et al 2008) and the Generalized Anxiety Disorder scale (GAD-7; Spitzer et al. 2006).

**Distal (pre-pandemic) risk factors**

We considered eleven distal (i.e., pre-pandemic) risk factors, assessed in the T1 survey**. Age** was assessed as a continuous variable and subsequently recategorized into 3 categories, i.e., 18-29/30-49/50-64/65< years for analysis. **Gender** was categorized as “*female*” and “*male*”. **Country of birth** was assessed using a dropdown list including all countries, and subsequently recategorized into “*Spain*” and “*other*” for analysis. **Marital status** was assessed with response options “*single*”, “*divorced or legally separated*”, “*widowed*”, and “*married*”, and subsequently recategorized into “*married*”, and “*single, divorced, separated, or widowed*” for analysis. **Pre-pandemic monthly income** was assessed using response options “*below 570€*” / “*570-799€*” / “*800-1,049€*” / “*1,050-1,299€*” / “*1,300-1,549€*” / “*1,550-1,799€*” / “*1,800-2,199€*” / “*2,200-2,699€*” / “*2,700-3,599€*” / “*3,600-4,499€*” / “*4,500-5,999€*” / “*over 6,000€*”, and subsequently recategorized into “*below 2,200€*”, “*2,200-4,500€*”, and “*over 4,500€*”. **Having children in care** was assessed as a dichotomy (no / yes). **Type of profession** was assessed as a checklist and subsequently recategorized into five groups for analysis: medical doctor; (2) nurse; (3) auxiliary nurse; (4) other profession involved in patient care (i.e., midwife, dentist or odontologist, pharmaceutical, laboratory, or radiology technician, psychologist, physiotherapist, social worker, patient transport); and (5) other profession not involved in patient care (i.e., administrative and management personnel, logistic support, research‐only personnel). **Type of workplace** was assessed as a checklist and subsequently categorized into four groups for analysis: (1) Emergency Department (ED) of a hospital, (2) Hospital (other than ED), (3) primary care, and (4) others (e.g., pharmacy, public health service, institution for people with disabilities). **Number of pre-pandemic lifetime mental disorders** was assessed using Composite International Diagnostic Interview (CIDI) 3·0 adapted screener items (Kessler & Üstün, 2004) including mood (i.e., depressive and bipolar disorders), anxiety (i.e., panic, generalized anxiety and obsessive-compulsive disorders), substance use (i.e., alcohol, illicit drugs, and prescription drugs with or without prescription), and other disorders. A sum score was created and categorized into “*none*”, “*exactly one*”, and “*two or more*”. **Number of pre-pandemic physical health conditions** was assessed using a 7-item checklist (Sangha et al. 2003) including respiratory diseases (not provoked by coronavirus), cardiovascular diseases, diabetes, cancer, chronic hepatic diseases, immunological diseases, and “other”. A sum score was created and categorized into “*none*”, “*exactly one*”, and “*two or more*”. **Twelve-month physical or sexual assault** was assessed as a dichotomy (no / yes).

**Proximal risk factors (pandemic-related stressful experiences)**

Four domains of pandemic-related stressful experiences were assessed in the T1 survey, using as time frame the onset of the virus outbreak in Spain. **A first domain** considered **three COVID-19 infection-related stressful experiences**: **(1)** **personal COVID-19 infection status** was categorized into “*having been hospitalized for COVID-19*”, “*positive COVID-19 test or medical COVID-19 diagnosis without hospitalization*”, and “*never infected with COVID-19*”; **(2)** **having loved ones infected with COVID‐19** was categorized into “*no loved ones infected*”, “*partner, children, or parents infected*”, “*other family, friends or others infected*”; and **(3)** **having been in isolation or quarantine because of COVID‐19** was assessed as a dichotomy (no/yes).

**The second domain** included **eight work-related stressful experiences**: **(1) average weekly hours worked** was assessed as a continuous variable an subsequently categorized into “*40 hours or less*”, “*41-50 hours*”, and “*51 hours or more*” for analysis; **(2) changes in assigned functions, team, or working location** was categorized into “*no changes*”, “*having changed of team or assigned functions (but not having changed to a specific COVID‐19‐related work location)*”, and “*having changed to a specific COVID‐19‐related work location (e.g., emergency room, COVID ward, fever clinic, intensive care unit, quarantine center, field hospital)*”; **(3) perceived lack of training for assigned tasks** was assessed using a 5-level Likert-type scale ranging from “*none of the time*” to “*all of the time*”; **(4) the frequency of direct exposure to COVID-19 patients** during professional activity was assessed using a 5-level Likert-type scale ranging from “*none of the time*” to “*all of the time*”; **(5) the perceived lack of healthcare center preparedness** was assessed four five-level Likert type scales that assessed lack of coordination, communication, personnel, and supervision at work, respectively (Cronbach α= 0.86). Each scale had response options from “*none of the time*” to “*all of the time*”. Items were summed and rescaled to a 0–4 summary scale score; **(6) perceived frequency of lack of protective equipment** was assessed using a 5-level Likert-type scale ranging from “*none of the time*” to “*all of the time*”; **(7) having to make decisions regarding prioritizing care among COVID‐19 patients** was assessed as a dichotomy (no/yes) among medical doctors and nurses; and **(8) having patient(s) in care that died from COVID‐19** was assessed as a dichotomy (no/yes) among all HCW involved in patient care.

**A third domain** consisted of **six variables that measured health-related stress** using five-level Likert type scales: **(1) feeling of little control over getting infected or not** was assessed using a 5-level Likert-type scale ranging from “*none of the time*” to “*all of the time*”; **(2) fear of infecting loved ones** was assessed using a 5-level Likert-type scale ranging from “*none of the time*” to “*all of the time*”; **(3)** **family and friends degree of worry of getting infected through the HCW** was assessed using a 5-level Likert-type scale ranging from “*none of the time*” to “*all of the time*”; **(4) degree to which people avoided** **the HCW’s family because of the HCW’s job** was assessed using a 5-level Likert-type scale ranging from “*none of the time*” to “*all of the time*”; **(5) personal health-related stress** was assessed using two 5-level Likert-type scales (concern about the respondent possibly being infected with COVID 19, concern about own respondents’ health in general; Cronbach α= 0.80). Items were summed and rescaled to a 0–4 scale score; and **(6) stress related to the health of loved ones** was assessed using two 5-level Likert-type scales (concern about loved ones being infected with COVID 19, concerns about the health of loved ones; Cronbach α= 0.85). Items were summed and rescaled to a 0–4 scale score.

**A fourth domain** consisted of **two financial stressful experiences**: **(1) having suffered a significant loss in personal or family income due to the COVID‐19 pandemic** was assessed as a dichotomy (no/yes); and **(2) stress over one's financial situation** was assessed using two 5-level Likert-type scales (financial stress, stress related to loss of job or income due to COVID-19; Cronbach α= 0.82). Items were summed and rescaled to a 0–4 scale score.

**References**

Blevins, C. A., Weathers, F. W., Davis, M. T., Witte, T. K., & Domino, J. L. (2015). The posttraumatic stress disorder checklist for DSM‐5 (PCL‐5): Development and initial psychometric evaluation. *Journal of Traumatic Stress*, 28(6), 489-498.

Bridgland, V. M. E., Moeck, E. K., Green, D. M., Swain, T. L., Nayda, D. M., Matson, L. A., Hutchison, N. P., & Takarangi, M. K. T. (2021). Why the COVID-19 pandemic is a traumatic stressor. *PLOS One*, 16(1), e0240146. <https://doi.org/10.1371/journal.pone.0240146>

Bryant, R. A. (2018). The Current Evidence for Acute Stress Disorder. *Current Psychiatry Reports* 20(12), 111. <https://doi.org/10.1007/s11920-018-0976-x>

Kessler, R. C., & Üstün, B. B. (2004). The World Mental Health (WMH) Survey Initiative Version of the World Health Organization (WHO) Composite International Diagnostic Interview (CIDI). International Journal of Methods in Psychiatric Research, 13(2), 93–117. <https://doi.org/10.1002/MPR.168>

Kroenke, K., Strine, T. W., Spitzer, R. L., Williams, J. B. W., Berry, J. T., Mokdad, A. H. (2009). The PHQ-8 as a measure of current depression in the general population. *Journal of Affective Disorders*, 114(1–3), 163–173. <https://doi.org/10.1016/j.jad.2008.06.026>

Sangha, O., Stucki, G., Liang, M. H., Fossel, A. H., & Katz, J. N. (2003). The Self-Administered Comorbidity Questionnaire: a new method to assess comorbidity for clinical and health services research. Arthritis and Rheumatism, 49(2), 156–163. <https://doi.org/10.1002/ART.10993>

Spitzer, R. L., Kroenke, K., Williams, J. B. W., Löwe, B. (2006). A Brief Measure for Assessing Generalized Anxiety Disorder. *Archives of Internal Medicine*, 166(10), 1092. <https://doi.org/10.1001/archinte.166.10.1092>

Weathers, F. W., Litz, B. T., Keane, T. M., Palmieri, P. A., Marx, B. P., & Schnurr, P. P. (2013). The PTSD Checklist for DSM-5 (PCL-5). National Center for PTSD. <https://doi.org/10.1037/t02622-000>

Zuromski, K. L., Ustun, B., Hwang, I., Keane, T. M., Marx, B. P., Stein, M. B., Ursano, R. J., & Kessler, R. C. (2019). Developing an optimal short‐form of the PTSD Checklist for DSM‐5 (PCL‐5). *Depression and Anxiety*, 36(9), 790–800. <https://doi.org/10.1002/da.22942>
