# Supplementary material for: Traumatic stress symptoms among Spanish healthcare workers during the COVID-19 pandemic: a prospective study
Source: Epidemiol Psychiatr Sci. 2023 Aug 9;32:e50. doi: 10.1017/S2045796023000628 (PMC10465320; doi:10.1017/S2045796023000628)
Supplement: Supplementary file 1 [file epssup.zip › S2045796023000628sup003.docx]

**Supplementary Table 1. Comparison of T1 and 4-month follow-up samples.**

|  | **Full T1 sample (n= 8,996)** | | **T1-only responders (n= 4,187)** | **T2 responders (n= 4,809)** |
| --- | --- | --- | --- | --- |
|  | **n^a^** | **% (SE)^b^** | **%^b^ (SE)** | **%^b^ (SE)** |
| **DISTAL (PRE-PANDEMIC) RISK FACTORS** |  |  |  |  |
| Age |  |  |  |  |
| - 18-29 years | 1,176 | 10.8 (1.7) | 13.3 (2.1) | 8.5 (1.3) |
| - 30-49 years | 4,197 | 45.8 (1.5) | 45.6 (1.8) | 46.1 (1.7) |
| - 50 years or more | 3,623 | 43.4 (2.7) | 41.1 (3.0) | 45.4 (2.5) |
| modified Rao-Scott (R-S) ꭓ^2^ test |  |  |  | 9.96 (3,00) [<0.001]* |
| Gender |  |  |  |  |
| - Male | 1,733 | 22.7 (1.3) | 22.7 (1.6) | 22.7 (1.2) |
| - Female | 7,263 | 77.3 (1.3) | 77.3 (1.6) | 77.3 (1.2) |
| modified Rao-Scott (R-S) ꭓ^2^ test |  |  |  | <0.001 (1,18E8) [0.97] |
| Country of birth |  |  |  |  |
| - Spain | 8,547 | 95.5 (0.5) | 95.3 (0.7) | 95.6 (0.4) |
| - Other | 449 | 4.5 (0.5) | 4.7 (0.7) | 4.4 (0.4) |
| modified Rao-Scott (R-S) ꭓ^2^ test |  |  |  | 0.29 (1,35) [0.59] |
| Marital status |  |  |  |  |
| - Married | 4,605 | 53.1 (2.1) | 51.7 (2.2) | 54.3 (2.1) |
| - Single, divorced, legally separated, or widowed | 4,392 | 46.9 (2.1) | 48.3 (2.2) | 45.7 (2.1) |
| modified Rao-Scott (R-S) ꭓ^2^ test |  |  |  | 3.99 (1,76) [0.05]* |
| Pre-pandemic monthly income |  |  |  |  |
| - Less than 2,200€ | 2,993 | 35.7 (2.2) | 40.2 (2.3) | 31.8 (2.1) |
| - Between 2,200€ - 4,500€ | 3,077 | 34.3 (1.0) | 31.7 (1.2) | 36.6 (1.1) |
| - More than 4,500€ | 2,926 | 29.9 (1.4) | 28.1 (1.7) | 31.5 (1.5) |
| modified Rao-Scott (R-S) ꭓ^2^ test |  |  |  | 16.41 (2,12) [<0.001]* |
| Having children in care |  |  |  |  |
| - No | 5,395 | 59.1 (1.5) | 60.4 (2.0) | 57.9 (1.3) |
| - Yes | 3,602 | 40.9 (1.5) | 39.6 (2.0) | 42.1 (1.3) |
| modified Rao-Scott (R-S) ꭓ^2^ test |  |  |  | 2.58 (1,26) [0.11] |
| Type of profession |  |  |  |  |
| - Medical doctor | 2,870 | 26.2 (2.8) | 23.8 (2.8) | 28.2 (2.9) |
| - Nurse | 2,660 | 30.8 (1.0) | 31.1 (1.1) | 30.5 (1.3) |
| - Auxiliary nurse | 874 | 13.6 (3.2) | 16.0 (3.5) | 11.4 (2.9) |
| - Other profession involved in patient care | 980 | 9.2 (0.8) | 8.1 (0.8) | 10.2 (0.9) |
| - Other profession not involved in patient care | 1,611 | 20.3 (2.1) | 21.0 (2.0) | 19.7 (2.4) |
| modified Rao-Scott (R-S) ꭓ^2^ test |  |  |  | 9.40 (4,22) [<0.001]* |
| Type of workplace |  |  |  |  |
| - Hospital (ED) | 5,457 | 57.6 (13.6) | 60.3 (12.9) | 55.1 (14.2) |
| - Hospital (not ED) | 2,737 | 35.6 (14.3) | 32.1 (13.6) | 38.6 (14.9) |
| - Primary Care | 802 | 6.9 (1.3) | 7.6 (1.3) | 6.2 (1.3) |
| - Others |  |  |  | 4.51 (2,42) [0.01]* |
| modified Rao-Scott (R-S) ꭓ^2^ test |  |  |  |  |
| Number of pre-pandemic lifetime mental disorders |  |  |  |  |
| - None | 5,380 | 59.5 (0.8) | 59.3 (0.9) | 59.6 (0.8) |
| - Exactly one | 2,900 | 32.4 (0.7) | 32.7 (1.1) | 32.2 (0.8) |
| - Two or more | 716 | 8.1 (0.4) | 8.0 (0.7) | 8.2 (0.8) |
| modified Rao-Scott (R-S) ꭓ^2^ test |  |  |  | 0.08 (2,27) [0.92] |
| Number of pre-pandemic physical health conditions |  |  |  |  |
| - None | 6,832 | 74.5 (0.8) | 75.3 (1.1) | 73.9 (1.0) |
| - Exactly one | 1,872 | 21.7 (0.7) | 21.2 (1.0) | 22.1 (0.8) |
| - Two or more | 292 | 3.8 (0.4) | 3.6 (0.4) | 4.0 (0.4) |
| modified Rao-Scott (R-S) ꭓ^2^ test |  |  |  | 0.78 (2,23) [0.46] |
| Twelve-month physical or sexual assault |  |  |  |  |
| - No | 8,883 | 98.7 (0.2) | 98.7 (0.3) | 98.7 (0.2) |
| - Yes | 113 | 1.3 (0.2) | 1.3 (0.3) | 1.3 (0.2) |
| modified Rao-Scott (R-S) ꭓ^2^ test |  |  |  | 0.06 (1,76) [0.81] |
| **PANDEMIC-RELATED STRESSFUL EXPERIENCES** |  |  |  |  |
| **A. COVID-19 INFECTION-RELATED STRESSFUL EXPERIENCES** |  |  |  |  |
| Personal COVID-19 infection status |  |  |  |  |
| - Not infected | 7,348 | 82.7 (2.2) | 84.0 (2.3) | 81.5 (2.3) |
| - Positive COVID-19 test or medical COVID-19 diagnosis without hospitalization | 1,539 | 16.1 (2.1) | 15.0 (2.1) | 17.2 (2.1) |
| - Having been hospitalized for COVID-19 | 109 | 1.2 (0.2) | 1.0 (0.2) | 1.4 (0.2) |
| modified Rao-Scott (R-S) ꭓ^2^ test |  |  |  | 4.14 (2,49) [0.02]* |
| Having loved ones infected with COVID-19 |  |  |  |  |
| - No loved ones infected | 2,172 | 27.6 (2.8) | 30.6 (3.4) | 25.1 (2.4) |
| - Partner, children, or parents infected | 1,371 | 13.9 (2.1) | 12.7 (2.2) | 14.9 (2.1) |
| - Other family, friends or others infected | 5,453 | 58.5 (1.0) | 56.7 (1.7) | 60.1 (0.8) |
| modified Rao-Scott (R-S) ꭓ^2^ test |  |  |  | 10.12 (2,51) [<0.001]* |
| Having been in isolation or quarantine because of COVID-19 |  |  |  |  |
| - No | 6,599 | 74.6 (1.8) | 75.6 (2.1) | 73.7 (1.7) |
| - Yes | 2,397 | 25.4 (1.8) | 24.4 (2.1) | 26.3 (1.7) |
| modified Rao-Scott (R-S) Χ2 test |  |  |  | 2.42 (1,40) [0.12] |
| **B. WORK-RELATED STRESSFUL EXPERIENCES** |  |  |  |  |
| Average weekly hours worked |  |  |  |  |
| - 40 hours or less | 5,437 | 63.3 (2.1) | 63.6 (2.1) | 63.1 (2.3) |
| - 41-50 hours | 2,102 | 21.5 (2.1) | 20.6 (2.2) | 22.3 (2.2) |
| - 51 hours or more | 1,457 | 15.2 (0.9) | 15.8 (1.5) | 14.6 (0.7) |
| modified Rao-Scott (R-S) ꭓ^2^ test |  |  |  | 1.12 (2,35) [0.33] |
| Changes in assigned functions, team, or working location |  |  |  |  |
| - No changes | 3,869 | 45.2 (1.5) | 44.7 (1.7) | 45.7 (1.5) |
| - Changed of team or assigned functions | 3,019 | 34.0 (3.2) | 33.2 (3.3) | 34.8 (3.2) |
| - Changed to specific COVID-19 related work location | 2,108 | 20.7 (3.5) | 22.1 (3.6) | 19.6 (3.6) |
| modified Rao-Scott (R-S) ꭓ^2^ test |  |  |  | 3.92 (2,67) [0.02]* |
| Perceived lack of training for assigned tasks (scale 0-4) |  |  |  |  |
| - 0 | 3,129 | 36.4 (2.3) | 37.6 (2.3) | 35.5 (2.4) |
| - 1 | 1,851 | 20.1 (0.7) | 17.8 (0.6) | 22.0 (1.2) |
| - 2 | 2,392 | 25.9 (1.4) | 26.4 (1.7) | 25.4 (1.4) |
| - 3 | 905 | 9.5 (0.6) | 9.1 (0.6) | 9.9 (0.7) |
| - 4 | 719 | 8.1 (0.5) | 9.1 (0.6) | 7.2 (0.7) |
| modified Rao-Scott (R-S) ꭓ^2^ test |  |  |  | 5.38 (4,38) [<0.001]* |
| Frequency of direct exposure to COVID-19 patients during professional activity (scale 0-4) |  |  |  |  |
| - 0 | 1,162 | 12.0 (2.1) | 12.5 (2.3) | 11.7 (1.9) |
| - 1 | 1,168 | 14.1 (1.5) | 14.6 (1.6) | 13.8 (1.5) |
| - 2 | 2,566 | 30.2 (2.7) | 28.1 (2.4) | 32.0 (2.9) |
| - 3 | 1,891 | 20.7 (2.0) | 20.5 (1.7) | 20.9 (2.4) |
| - 4 | 2,209 | 22.9 (3.2) | 24.3 (3.3) | 21.6 (3.2) |
| modified Rao-Scott (R-S) ꭓ^2^ test |  |  |  | 3.31 (4,37) [0.01]* |
| Perceived lack of healthcare center preparedness (scaled 0-4) |  |  |  |  |
| - 0 | 669 | 8.6 (1.4) | 8.8 (1.6) | 8.5 (1.4) |
| - 1 | 1,842 | 21.3 (2.1) | 20.0 (1.7) | 22.5 (2.6) |
| - 2 | 3,302 | 35.7 (0.7) | 37.4 (0.9) | 34.3 (0.8) |
| - 3 | 2,339 | 25.3 (2.0) | 25.0 (2.0) | 25.5 (2.2) |
| - 4 | 845 | 9.0 (1.1) | 8.7 (1.0) | 9.3 (1.4) |
| modified Rao-Scott (R-S) ꭓ^2^ test |  |  |  | 1.96 (5,00) [0.10] |
| Perceived frequency of lack of protective equipment (scale 0-4) |  |  |  |  |
| - 0 | 982 | 10.8 (1.3) | 11.0 (1.5) | 10.6 (1.3) |
| - 1 | 1,243 | 14.4 (1.0) | 14.3 (1.4) | 14.5 (0.9) |
| - 2 | 3,315 | 36.7 (1.1) | 35.8 (1.1) | 37.5 (1.5) |
| - 3 | 2,336 | 25.5 (1.6) | 25.7 (1.7) | 25.4 (1.7) |
| - 4 | 1,120 | 12.5 (1.0) | 13.2 (1.3) | 12.0 (1.0) |
| modified Rao-Scott (R-S) ꭓ^2^ test |  |  |  | 0.57 (4,28) [0.68] |
| Having to make decisions regarding prioritizing care among COVID-19 patients |  |  |  |  |
| No | 7,370 | 83.6 (1.6) | 83.8 (1.6) | 83.5 (1.8) |
| Yes | 1,626 | 16.4 (1.6) | 16.2 (1.6) | 16.5 (1.8) |
| modified Rao-Scott (R-S) ꭓ^2^ test |  |  |  | 0.15 (1,36) [0.70] |
| Having patient(s) in care that died from COVID-19 |  |  |  |  |
| No | 5,466 | 63.1 (3.0) | 63.6 (2.9) | 62.7 (3.2) |
| Yes | 3,530 | 36.9 (3.0) | 36.4 (2.9) | 37.3 (3.2) |
| modified Rao-Scott (R-S) ꭓ^2^ test |  |  |  | 0.38 (1,34) [0.54] |
| **C. HEALTH-RELATED STRESSFUL EXPERIENCES** |  |  |  |  |
| Feeling of little control of getting infected or not (scale 0-4) |  |  |  |  |
| - 0 | 699 | 8.6 (0.8) | 9.1 (1.1) | 8.2 (0.8) |
| - 1 | 1,413 | 15.9 (1.0) | 14.7 (1.0) | 17.0 (1.1) |
| - 2 | 3,459 | 37.6 (0.9) | 37.5 (0.9) | 37.6 (1.3) |
| - 3 | 2,394 | 26.2 (1.4) | 25.7 (1.4) | 26.7 (1.5) |
| - 4 | 1,031 | 11.7 (0.9) | 13.1 (1.0) | 10.5 (0.9) |
| modified Rao-Scott (R-S) ꭓ^2^ test |  |  |  | 4.23 (4,14) [<0.001]* |
| Fear of infecting loved ones (scale 0-4) |  |  |  |  |
| - 0 | 307 | 3.6 (0.4) | 3.6 (0.5) | 3.6 (0.4) |
| - 1 | 638 | 7.0 (0.5) | 6.9 (0.7) | 7.1 (0.5) |
| - 2 | 1,825 | 20.5 (1.3) | 19.4 (1.3) | 21.5 (1.5) |
| - 3 | 2,096 | 22.6 (0.6) | 21.0 (0.8) | 24.0 (0.7) |
| - 4 | 4,130 | 46.3 (2.0) | 49.1 (2.3) | 43.9 (1.9) |
| modified Rao-Scott (R-S) ꭓ^2^ test |  |  |  | 4.02 (4,26) [<0.001]* |
| Family and friends’ degree of worry of getting infected through the HCW (scale 0-4) |  |  |  |  |
| - 0 | 1,355 | 15.4 (1.1) | 15.5 (1.1) | 15.4 (1.3) |
| - 1 | 1,778 | 19.3 (0.8) | 17.9 (1.0) | 20.6 (0.9) |
| - 2 | 3,004 | 33.3 (0.6) | 33.6 (0.9) | 33.1 (0.9) |
| - 3 | 1,616 | 17.3 (1.1) | 17.4 (1.4) | 17.3 (1.3) |
| - 4 | 1,243 | 14.6 (0.8) | 15.6 (1.1) | 13.7 (1.0) |
| modified Rao-Scott (R-S) ꭓ^2^ test |  |  |  | 1.71 (4,55) [0.14] |
| Degree to which people avoided the HCW’s family because of the HCW’s job (scale 0-4) |  |  |  |  |
| - 0 | 195 | 2.4 (0.2) | 2.4 (0.3) | 2.3 (0.2) |
| - 1 | 294 | 3.2 (0.4) | 3.7 (0.5) | 2.8 (0.4) |
| - 2 | 903 | 10.6 (0.8) | 10.1 (0.8) | 11.1 (1.1) |
| - 3 | 1,255 | 13.9 (0.8) | 13.9 (0.8) | 14.0 (1.0) |
| - 4 | 6,349 | 69.8 (1.2) | 69.8 (1.4) | 69.9 (1.2) |
| modified Rao-Scott (R-S) ꭓ^2^ test |  |  |  | 0.95 (4,5516) [0.44] |
| Personal health-related stress (scale 0-4) |  |  |  |  |
| - 0 | 451 | 5.5 (0.6) | 5.0 (0.6) | 6.0 (0.8) |
| - 1 | 2,395 | 25.5 (1.1) | 24.1 (1.4) | 26.8 (1.2) |
| - 2 | 3,159 | 34.8 (0.8) | 34.8 (1.0) | 34.8 (0.8) |
| - 3 | 2,158 | 24.3 (0.9) | 25.8 (1.3) | 23.0 (0.8) |
| - 4 | 833 | 9.8 (1.0) | 10.3 (1.1) | 9.4 (0.9) |
| modified Rao-Scott (R-S) ꭓ^2^ test |  |  |  | 4.04 (4,21) [<0.001]* |
| Stress related to the health of loved ones (scale 0-4) |  |  |  |  |
| - 0 | 72 | 1.0 (0.2) | 0.9 (0.3) | 1.1 (0.3) |
| - 1 | 778 | 9.3 (1.1) | 8.8 (1.1) | 9.8 (1.1) |
| - 2 | 2,435 | 26.7 (1.2) | 24.7 (1.4) | 28.3 (1.2) |
| - 3 | 2,835 | 31.2 (0.5) | 33.9 (0.8) | 28.9 (0.6) |
| - 4 | 2,875 | 31.8 (2.0) | 31.7 (2.0) | 31.8 (2.1) |
| modified Rao-Scott (R-S) ꭓ^2^ test |  |  |  | 6.46 (4,96) [<0.001]* |
| **D. FINANCIAL STRESSFUL EXPERIENCES** |  |  |  |  |
| Significant loss of personal or familial income due to COVID-19 |  |  |  |  |
| - No | 7,259 | 80.4 (0.7) | 79.6 (0.7) | 81.0 (1.0) |
| - Yes | 1,738 | 19.6 (0.7) | 20.4 (0.7) | 19.0 (1.0) |
| modified Rao-Scott (R-S) ꭓ^2^ test |  |  |  | 1.94 (1,10) [0.16] |
| Financial stress (scale 0-4) |  |  |  |  |
| - 0 | 2,719 | 29.4 (0.8) | 26.0 (1.0) | 32.3 (0.9) |
| - 1 | 3,038 | 33.1 (0.7) | 33.3 (0.9) | 32.9 (0.7) |
| - 2 | 1,840 | 21.1 (0.6) | 22.3 (0.7) | 20.1 (0.8) |
| - 3 | 955 | 11.2 (0.6) | 12.1 (0.8) | 10.4 (0.6) |
| - 4 | 445 | 5.2 (0.5) | 6.3 (0.6) | 4.3 (0.4) |
| modified Rao-Scott (R-S) ꭓ^2^ test |  |  |  | 15.44 (4,58) [<0.001]* |

Abbreviations: ED = emergency department; SE= standard error.

a Number of observations (n) are unweighted.

b Proportions (%, SE) are weighted.

**Supplementary Table 2. Associations of pandemic-related stressful experiences with traumatic stress symptoms, additionally adjusting for co-occurring depression and anxiety (n= 4,809).**

|  | **Sample descriptives** | | **T1 prevalence TSS^a^** | **Incidence TSS^b^** | **Persistence TSS^c^** |
| --- | --- | --- | --- | --- | --- |
|  | **n^d^** | **% (SE) or Med (SE) (IQR)^e^** | **OR (95% CI)^f^** | **OR (95% CI)^f^** | **OR (95% CI)^f^** |
| **A. COVID-19 infection-related stressful experiences** |  |  |  |  |  |
| Personal COVID-19 infection status |  |  |  |  |  |
| - Never infected with COVID-19 | 3,873 | 82.5 (2.1) | (ref) | (ref) | (ref) |
| - Positive COVID-19 test or medical COVID-19 diagnosis without hospitalization | 869 | 16.1 (2.0) | 0.90 (0.67-1.20) | 1.16 (0.81-1.66) | 0.83 (0.56-1.22) |
| - Having been hospitalized for COVID-19 | 67 | 1.4 (0.2) | 0.71 (0.30-1.69) | 0.81 (0.24-2.76) | 0.35 (0.06-2.03) |
| Having loved ones infected with COVID-19 |  |  |  |  |  |
| - No loved ones infected | 1,065 | 27.7 (2.4) | (ref) | (ref) | (ref) |
| - Partner, children, or parents infected | 781 | 13.7 (2.0) | 0.76 (0.47-1.25) | 1.24 (0.91-1.68) | 1.03 (0.56-1.90) |
| - Other family, friends or others infected | 2,964 | 58.6 (0.9) | 0.79 (0.59-1.05) | 0.94 (0.63-1.40) | 1.12 (0.77-1.62) |
| Having been in isolation or quarantine because of COVID-19 |  |  |  |  |  |
| - No | 3,472 | 74.4 (1.7) | (ref) | (ref) | (ref) |
| - Yes | 1,337 | 25.6 (1.7) | 0.96 (0.80-1.15) | 1.16 (0.83-1.63) | 1.31 (0.86-2.00) |
| **B. Work-related stressful experiences** |  |  |  |  |  |
| Average weekly hours worked |  |  |  |  |  |
| - 40 hours or less | 2,905 | 63.2 (2.5) | (ref) | (ref) | (ref) |
| - 41-50 hours | 1,140 | 22.7 (2.4) | 1.02 (0.82-1.26) | 0.72 (0.46-1.12) | 0.91 (0.52-1.60) |
| - 51 hours or more | 765 | 14.0 (0.7) | 1.11 (0.80-1.54) | 0.82 (0.49-1.38) | 0.97 (0.54-1.71) |
| Changes in assigned functions, team, or working location |  |  |  |  |  |
| - No changes | 2,088 | 45.3 (1.6) | (ref) | (ref) | (ref) |
| - Changed of team or assigned functions | 1,642 | 33.7 (3.2) | 1.49 (1.16-1.90)* | 1.38 (0.86-2.22) | 1.37 (0.93-2.01) |
| - Changed to specific COVID-19 related work location | 1,080 | 21.1 (3.7) | 1.76 (1.37-2.26)* | 1.67 (0.96-2.92) | 1.25 (0.71-2.19) |
| Perceived lack of training for assigned tasks (scale 0-4) |  | 0.7 (0.1) (0.0-1.7) | 1.21 (1.10-1.33)* | 1.00 (0.88-1.13) | 1.06 (0.91-1.23) |
| Frequency of direct exposure to COVID-19 patients during professional activity (scale 0-4) |  | 1.8 (0.2) (1.0-2.9) | 1.19 (1.08-1.30)* | 1.20 (0.99-1.46) | 1.17 (0.99-1.38) |
| Perceived lack of healthcare center preparedness (scaled 0-4) |  | 1.6 (0.1) (0.7-2.4) | 1.24 (1.09-1.40)* | 1.32 (1.13-1.54)* | 1.05 (0.84-1.32) |
| Perceived frequency of lack of protective equipment (scale 0-4) |  | 1.7 (0.1) (1.0-2.5) | 1.47 (1.35-1.61)* | 1.22 (1.05-1.41)* | 1.04 (0.82-1.32) |
| Having to make decisions regarding prioritizing care among COVID-19 patients |  |  |  |  |  |
| - No | 3,919 | 84.2 (1.8) | (ref) | (ref) | (ref) |
| - Yes | 890 | 15.8 (1.8) | 1.20 (0.92-1.58) | 1.29 (0.88-1.89) | 0.86 (0.52-1.43) |
| Having patient(s) in care that died from COVID-19 |  |  |  |  |  |
| - No | 2,882 | 62.8 (3.2) | (ref) | (ref) | (ref) |
| - Yes | 1,927 | 37.2 (3.2) | 1.04 (0.78-1.39) | 1.29 (0.86-1.95) | 1.86 (1.14-3.03)* |
| **C. Health-related stressful experiences** |  |  |  |  |  |
| Feeling of little control of getting infected or not (scale 0-4) |  | 1.7 (0.1) (1.0-2.5) | 1.33 (1.16-1.51)* | 1.21 (1.12-1.31)* | 0.95 (0.75-1.21) |
| Fear of infecting loved ones (scale 0-4) |  | 2.8 (0.1) (1.7-3.4) | 1.18 (0.96-1.44) | 1.40 (1.21-1.61)* | 1.03 (0.79-1.34) |
| Family and friends’ degree of worry of getting infected through the HCW (scale 0-4) |  | 1.5 (0.1) (0.5-2.4) | 1.21 (1.10-1.33)* | 1.11 (0.98-1.26) | 0.96 (0.79-1.17) |
| Degree to which people avoided the HCW’s family because of the HCW’s job (scale 0-4) |  | 0.0 (0.0) (0.0-0.4) | 1.25 (1.13-1.38)* | 1.14 (1.02-1.27)* | 1.13 (0.92-1.38) |
| Personal health-related stress (scale 0-4) |  | 1.6 (0.1) (0.8-2.4) | 1.53 (1.37-1.72)* | 1.32 (1.16-1.50)* | 0.99 (0.79-1.24) |
| Stress related to the health of loved ones (scale 0-4) |  | 2.4 (0.1) (1.5-3.2) | 1.52 (1.32-1.75)* | 1.41 (1.21-1.65)* | 1.07 (0.79-1.46) |
| **D. Financial stressful experiences** |  |  |  |  |  |
| Significant loss of personal or familial income due to COVID-19 |  |  |  |  |  |
| - No | 3,924 | 79.8 (1.2) | (ref) | (ref) | (ref) |
| - Yes | 885 | 20.2 (1.2) | 1.17 (0.86-1.58) | 1.61 (1.15-2.26)* | 1.10 (0.62-1.94) |
| Financial stress (scale 0-4) |  | 0.6 (0.0) (0.0-1.6) | 1.24 (1.15-1.34)* | 1.13 (0.99-1.29) | 1.04 (0.90-1.21) |

Abbreviations: CI = confidence interval; ED = emergency department; IQR=interquartile range; Med= median; OR = odds ratio; SE = standard error; TSS = traumatic stress symptoms.

a. Prevalence of TSS is defined as a positive screen on the 4-item PCL-5 at T1 (n = 4,809).

b. Incidence of TSS is defined as the proportion of respondents with a positive 4-item PCL-5 screen at four-month follow-up (n = 412) among those with a negative 4-item PCL-5 screen at T1 (n = 3,796).

c. Persistence of TSS is defined as the proportion of respondents with a positive 4-item PCL-5 screen at four-month follow-up (n = 536) among those with a positive 4-item PCL-5 screen at T1 (n = 1,013).

d. Number of observations (n) are unweighted.

e. Proportions (%, SE) and Medians (SE) (IQR) are weighted.

f. Each row represents a separate logistic regression model, each time adjusting for all distal risk factors, time (i.e., week) of T1 survey participation, and co-occurring depression (PHQ-8 total scale score) and anxiety (GAD-7 total scale score). Analyses of T1 TSS prevalence are adjusted for T1 PHQ-8 and T1 GAD-7 total scale scores; analyses of TSS incidence and persistence are adjusted for 4-month PHQ-8 and 4-month GAD-7 total scale scores.

* Indicate statistically significant results (α=0.05).

**Supplementary Table 3. Population Attributable Risk Proportions (PARP) for the associations of pandemic-related stressful experiences with TSS, additionally adjusting for co-occurring depression and anxiety (n= 4,809).**

|  | **T1 prevalence TSS^a^** | **Incidence TSS^b^** | **Persistence TSS^c^** |
| --- | --- | --- | --- |
|  | **% (SE)^d,e^** | **% (SE)^d,e^** | **% (SE)^d,e^** |
| **A. COVID-19 infection-related stressful experiences** |  |  |  |
| - Personal COVID-19 infection | -1.0 (1.3) | 1.6 (2.3) | -1.6 (1.6) |
| - Having loved ones infected with COVID-19 | -8.1 (4.9) | -0.2 (8.1) | 2.1 (5.8) |
| - Having been in isolation or quarantine because of COVID-19 | -0.5 (1.7) | 2.9 (3.1) | 2.2 (2.0) |
| Risk domain A. - total PARP^f^ | -8.8 (5.2) | 1.5 (8.1) | 2.1 (6.0) |
| **B. Work-related stressful experiences** |  |  |  |
| - Average weekly hours worked | 0.9 (2.4) | -6.1 (3.4) | -0.9 (3.0) |
| - Changes in assigned functions, team, or working location | 11.2 (2.9)* | 12.8 (5.5)* | 5.0 (4.0) |
| - Perceived lack of training for assigned tasks (scale 0-4) | 12.2 (3.1)* | -0.3 (5.1) | 2.9 (4.7) |
| - Frequency of direct exposure to COVID-19 patients during professional activity (scale 0-4) | 16.8 (5.2)* | 22.7 (8.9)* | 11.7 (8.1) |
| - Perceived lack of healthcare center preparedness (rescaled 0-4) | 19.2 (5.4)* | 30.8 (8.5)* | 3.7 (7.5) |
| - Perceived frequency of lack of protective equipment (scale 0-4) | 33.7 (4.5)* | 22.4 (7.8)* | 3.1 (7.3) |
| - Having to make decisions regarding prioritizing care among COVID-19 patients | 1.4 (1.5) | 2.9 (2.2) | -0.9 (1.8) |
| - Having patient(s) in care that died from COVID-19 | 0.7 (2.5) | 5.9 (4.1) | 8.0 (3.0)* |
| Risk domain B. - total PARP^f^ | 45.5 (5.9)* | 46.4 (10.4)* | 14.6 (10.1) |
| **C. Health-related stressful experiences** |  |  |  |
| - Feeling of little control of getting infected or not (scale 0-4) | 26.1 (5.4)* | 23.1 (8.5)* | -3.7 (8.4) |
| - Fear of infecting loved ones (scale 0-4) | 20.7 (8.3)* | 47.6 (9.4)* | 2.4 (11.3) |
| - Family and friends’ degree of worry of getting infected through the HCW (scale 0-4) | 16.5 (4.9)* | 11.2 (7.1) | -2.9 (6.6) |
| - Degree to which people avoided the HCW’s family because of the HCW’s job (scale 0-4) | 6.0 (1.6)* | 4.2 (2.8) | 2.9 (2.4) |
| - Personal health-related stress (scale 0-4) | 37.5 (5.0)* | 30.1 (8.6)* | -1.0 (7.8) |
| - Stress related to the health of loved ones (scale 0-4) | 46.8 (6.4)* | 46.6 (9.6)* | 7.0 (11.5) |
| Risk domain C. - total PARP^f^ | 52.0 (7.2)* | 59.1 (9.3)* | 1.8 (13.6) |
| **D. Financial stressful experiences** |  |  |  |
| - Significant loss of personal or familial income due to COVID-19 | 1.3 (1.3) | 5.4 (2.3)* | 0.6 (1.7) |
| - Financial stress (scale 0-4) | 12.1 (3.1)* | 8.7 (4.7) | 1.7 (4.2) |
| Risk domain D. - total PARP^f^ | 12.1 (3.1)* | 8.7 (4.7) | 1.6 (4.1) |

Abbreviations: COVID-19 = coronavirus disease 2019; SE = standard error; TSS = traumatic stress symptoms.

a. Prevalence of TSS is defined as a positive screen on the 4-item PCL-5 at T1 (n = 4,809).

b. Incidence of TSS is defined as the proportion of respondents with a positive 4-item PCL-5 screen at four-month follow-up (n = 412) among those with a negative 4-item PCL-5 screen at T1 (n = 3,796).

c. Persistence of TSS is defined as the proportion of respondents with a positive 4-item PCL-5 screen at four-month follow-up (n = 536) among those with a positive 4-item PCL-5 screen at T1 (n = 1,013).

d. Proportions (%, SE) are weighted.

e. Each row represents a separate logistic regression model, each time adjusting for all distal risk factors and time (i.e., week) of T1 survey participation.

f. Risk domain total PARPs are based on four separate logistic regression models, one for each of the four proximal risk factor domains (A-D). Each model includes the proximal risk factors from the corresponding proximal risk factor domain (but not the other domains), adjusting for all distal risk factors, time (i.e., week) of T1 survey participation, and co-occurring depression (PHQ-8 total scale score) and anxiety (GAD-7 total scale score). Analyses of T1 TSS prevalence are adjusted for T1 PHQ-8 and T1 GAD-7 total scale scores; analyses of TSS incidence and persistence are adjusted for 4-month PHQ-8 and 4-month GAD-7 total scale scores.

* Indicate statistically significant results (α=0.05).
